# Targeted Enrichment and Sequencing of Recent Endosymbiont-Host Lateral Gene Transfers

**DOI:** 10.1038/s41598-017-00814-4

**Published:** 2017-04-12

**Authors:** Julie C. Dunning Hotopp, Barton E. Slatko, Jeremy M. Foster

**Affiliations:** 1 Institute for Genome Sciences, Department of Microbiology and Immunology, University of Maryland, Baltimore, MD 21201 United States; 2 Genome Biology Division, New England Biolabs, Inc., 240 County Road, Ipswich, MA 01938 United States

## Abstract

Lateral gene transfer (LGT) from microbial symbionts to invertebrate animals is described at an increasing rate, particularly between *Wolbachia* endosymbionts and their diverse invertebrate hosts. We sought to assess the use of a capture system to cost-effectively sequence such LGT from the host genome. The sequencing depth of Illumina paired end data obtained with a *Wolbachia* capture system correlated well with that for an Illumina paired end data set used to detect LGT in *Wolbachia*-depleted *B. malayi* (p-value: <2e-16). Using a sequencing depth threshold of two or three standard deviations above the mean, 96.9% or 96.7% of positions, respectively, are predicted in the same manner between the two datasets, with 24.7% or 42.5% of the known 49.0 kbp of LGT sequence predicted correctly, respectively. Prior qPCR results for nuwts showed similar correlations for both datasets supporting our conclusion that oligonucleotide-based capture methods can be used to obtain sequences from *Wolbachia-*host LGT. However, at least 121 positions had a minority of the reads supporting the endosymbiont reference base call using the capture data, illustrating that sequence reads from endosymbiont-host LGTs can confound endosymbiont genome projects, erroneously altering the called consensus genome, a problem that is irrespective to the sequencing technology or platform.

## Introduction

The transfer of DNA between diverse organisms through lateral gene transfer (LGT) can allow organisms to acquire novel functional genes. Although most described LGT events occur within a single domain of life, LGT of functional genes or genetic elements has also been described between different domains of life such as bacteria and eukaryotes, including animals. For example, *Bartonella henselae* can naturally transfer its plasmid via the Type IV secretion system to human cells^1^. Bdelloid rotifers (small freshwater invertebrates that lack sexual reproduction) contain DNA from bacteria, fungi, and plants^2^. *Hypothenemus hampei*, the coffee berry borer, acquired a *Bacillus* mannanase gene^3^. Likewise, several plant parasitic nematodes have acquired plant cell wall-degrading enzymes from bacteria including cellulases that allow the nematodes to invade plant tissues^4–6^.

Numerous cases of LGT have been detected between *Wolbachia* endosymbionts and the genomes of diverse invertebrate taxa that are the hosts of these endosymbionts^7–22^. *Wolbachia* endosymbionts colonize a wide range of arthropods and filarial nematodes, including ~40% of insect species^23–25^. Like mitochondria, *Wolbachia* cells are maternally inherited and transferred through the egg cytoplasm^23, 24^, providing ample opportunity for LGT of bacterial genes to eukaryotic genomes. We have adopted the nomenclature of nuwts for such nuclear *Wolbachia* transfers following the existing standard for nuclear mitochondrial transfers (numts) and nuclear plastid transfers (nupts).

In 2001, Kondo *et al*. first described a nuwt in the bean beetle *Callosobruchus chinensis* using X-linked inheritance and inverse PCR^12^. Numerous *Wolbachia* genomes have been integrated into chromosome 4 of the *D. ananassae* Hawaii 2L chromosome constituting >2% of the fly genome and 20% of chromosome 4^26^, with at least 28 *D. ananassae* genes of *Wolbachia* origin being transcribed, albeit at low levels^7^. Nuwts were detected in four lines of *D. ananassae* from Asia and the Pacific indicating that the nuwt in *D. ananassae* may be widely distributed^7^. In 2007, most (8/11) of the genome sequencing projects of invertebrates that harbored *Wolbachia* showed evidence of having LGT between the endosymbiont genome and the host chromosomes^7^ suggesting that nuwts are common. Nuwts were experimentally confirmed in all five of the hosts examined further^7^.

In nematodes, nuwts have been widely identified. One stronglyoidean nematode^22^ and all filarial nematodes examined to date have nuwts including *Brugia malayi* ^7^, *Brugia pahangi* ^7^, *Brugia timori* ^7^, *Acanthocheilonema viteae* ^14^, *Acanthocheilonema spirocauda* ^27^, *Onchocerca volvulus* ^8^, *Onchocerca flexuosa* ^14^, *Dirofilaria immitis* ^7^, and *Loa loa* ^28^. The stronglyoidean nematode as well as four filarial nematodes have nuwts despite currently lacking the *Wolbachia* endosymbiont^14, 22, 28^, demonstrating that these nematodes were once infected and have lost their endosymbiont. In filarial nematodes, one nuwt likely predates the divergence of *O. volvulus* and *Onchocerca ochengi* ^8^ while another predates the divergence of *B. malayi, B. pahangi*, and *B. timori* ^7^. In *O. flexuosa*, a nematode that today lacks *Wolbachia*, 97 nuwts were identified through transcriptome sequencing with some nuwt sequences being co-transcribed with nematode genes^29^. Proteomic analysis resulted in the identification of three peptides that map to two *Wolbachia* ABC transport-related proteins^29^. In a subsequent study, anti-sense probes to nuwt transcripts coding for HlyD, aminopeptidase P, and a hypothetical protein showed *in situ* hybridization based-labeling of the lateral chords and intestine of both sexes, the hypodermis, the empty uteri of young females, and the testis and developing sperm of males, while sense probes (negative controls) showed no labeling^30^. Immunohistochemical labeling of worm sections by antibodies raised against these peptides gave broadly similar results, indicative of translation^30^.

Despite these observations, detection of nuwts in filarial nematode genomes is still not routine. In arthropods, where *Wolbachia* is not an obligate mutualist, nuwts can be identified by sequencing insects treated with an antibiotic for multiple generations, such that the *Wolbachia* endosymbiont is eliminated and sequences generated are from the nuwts. However, this is not possible in filarial nematodes where *Wolbachia* endosymbionts are essential for nematode survival. In a model filarial nematode system, *Brugia malayi*, we demonstrated that nuwts could be detected from DNA collected from nematodes prior to the worm dying from an antibiotic treatment used to severely deplete *Wolbachia* abundance^17^, but this depletion strategy is not possible in all filarial systems as many are not routinely cultured in the laboratory.

Capture-based whole genome sequencing of *Wolbachia* endosymbionts has been successfully demonstrated for *Wolbachia* endosymbionts of filarial nematodes^31^ and arthropods^31^ and their phage^32^. *Wolbachia* genomes range in size from ~0.9–1.5 Mb. The generally smaller genome sizes of *Wolbachia* from nematodes is largely due to an absence of phage WO sequences, a lower level of repetitive DNA and a small number of gene losses^33^. To account for different genome sizes and DNA content across the most intensively studied *Wolbachia* supergroups, capture baits were designed on 11 complete *Wolbachia* genome sequences from supergroups A, B, C, and D available in the NCBI databases at the time of design. Approximately 215,000 oligonucleotide baits (120-mers) were tiled across these genome sequences with 60 base overlaps^31^. The entire genome of *w*Bm, the *Wolbachia* endosymbiont of *B. malayi* that was used in the capture bait design, was captured when total *B. malayi* DNA was used as a test case^31^. Similarly, >95% of the *Wolbachia* DNA was captured from total DNA isolated from the pill bug *Armadillidium vulgare*, which contains an endosymbiont whose genome was not used in the design^31^.

Here, we present data that the baits also capture nuwts through a comparison of two existing, published Illumina paired-end data sets for *B. malayi* ^17, 31^. The first data set, which is referred to as the depletion data, includes >138 million reads from a paired end library constructed directly from DNA from tetracycline-treated, and thus *Wolbachia-*depleted, *B. malayi* filarial nematodes^17^ (SRX142902). In this way, it is a gold standard for detecting nuwts in *B. malayi* since the *Wolbachia* endosymbiont genome is depleted to every extent possible, enabling the accurate detection of nuwts. The second data set, which is referred to as the capture data, includes >91 million reads from a paired end library constructed from *B. malayi* DNA where the *Wolbachia* endosymbionts were not depleted, but *Wolbachia* sequences were selectively sequenced after capture using Agilent Sure Select RNA baits (SRX1057997), as previously described^31^. The capture system enables a greater recovery of *Wolbachia* reads from both the endosymbiont genome and nuwts in the host genome. Such enrichment of *Wolbachia* sequences leads to a decrease in sequencing cost and/or an increase in sequencing depth. The capture dataset used here contains the *Wolbachia* sequences from both the endosymbiont and nematode genomes, which were sequenced simultaneously, but reads could alternatively be captured from endosymbiont*-*depleted samples in insects. While *Wolbachia* levels can sometimes be depleted, in many cases the levels of *Wolbachia* endosymbionts cannot be manipulated, and therefore the capture data set is derived from a DNA sample that represents a frequent DNA sample in filarial nematode genomics.

We rely on sequencing depth to identify and compare nuwts in both data sets as described in prior published work on *B. malayi* nuwts, where a subset were validated by qPCR and an analysis of SNPs^17^. Using this approach we are able to identify some, but not all, nuwts. We also find that nuwt sequences can inadvertently alter the consensus genome sequence of the endosymbiont relative to the actual genome sequence of the endosymbiont, a problem not unique to the use of a capture-based system.

## Results

### Identifying nuwts in the presence of sequences from a *Wolbachia* endosymbiont genome

**Figure 1:**
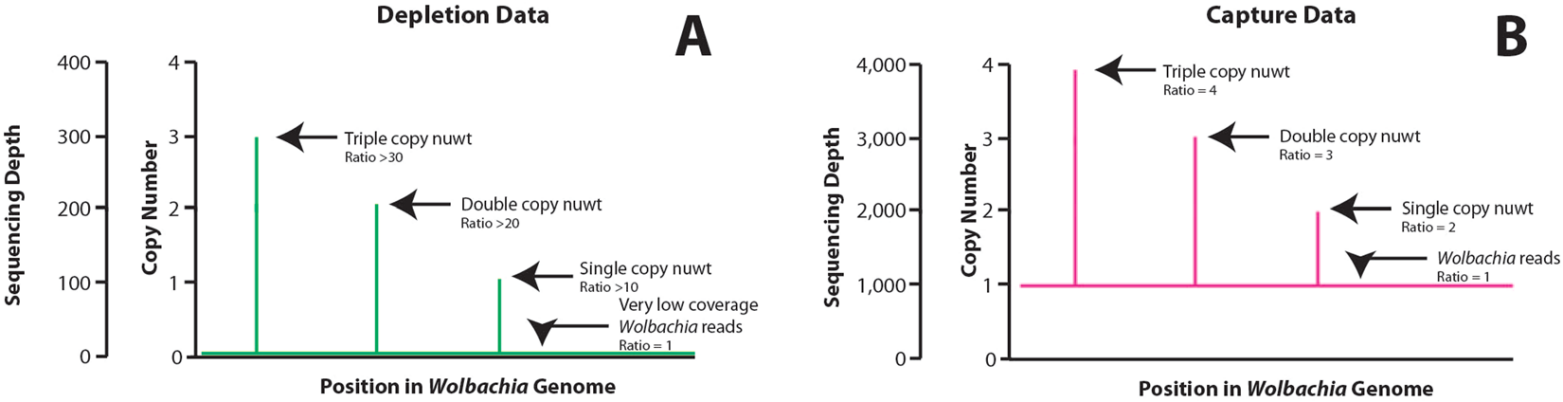
Schematic Depiction of Theoretical Differences Between the Capture and Depletion Data. This schematic depicts the differences between (**A**) a theoretical depletion data set and (**B**) a theoretical capture data set. The depletion data (**A**) is depicted here as low sequencing depth (<10X depth) on the *Wolbachia* genome and 100X depth on a typical single copy *Brugia* gene. Therefore, a single copy nuwt would have 100X depth while a nuwt with 2 or 3 copies would have 200X or 300X depth, respectively. The ratio of the depth for 1, 2, or 3 copy nuwts relative to *Wolbachia* endosymbiont DNA would be >10, >20, or >30 respectively. The capture data (**B**) is depicted here as having the same sequencing depth for the *Wolbachia* genome and a typical single copy *Brugia* gene (1000X depth). Therefore, a single copy nuwt would have 2000X depth while a nuwt with 2 or 3 copies would have 3000X or 400X depth, respectively. The ratio of the depth for 1, 2, or 3 copy nuwts relative to *Wolbachia* endosymbiont DNA would be 2, 3, or 4 respectively. This illustrates the effects of two important factors in the actual datasets examined in this study: (a) absolute sequencing depth (where capture $\gg$ depletion) and (b) the ratio of the sequencing depth for a *Wolbachia* sequence found as a nuwt relative to the same sequence found in the endosymbiont genome (where depletion $\gg$ capture).

**Figure 2:**
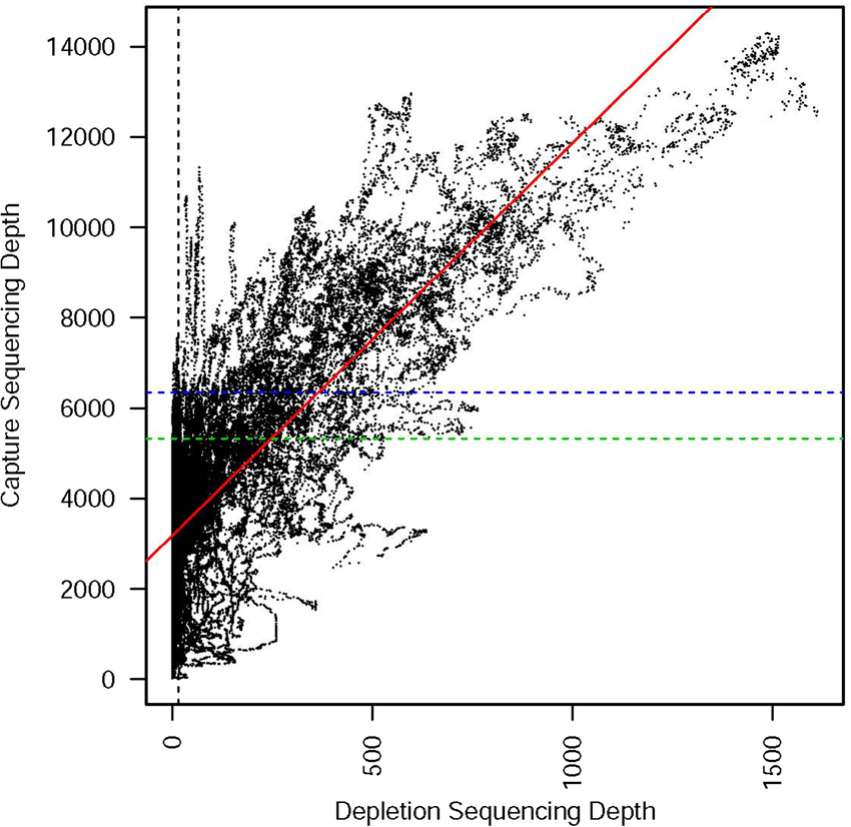
Scatterplot of Sequencing Depth Measurements for Capture and Depletion Data. The sequencing depth values for the capture data and the depletion data are plotted for every position in the reference genome. As expected, there is a positive linear correlation between the two values (red line, p-value: <2e-16, R-squared = 0.307). Our thresholds for predicting nuwts are overlaid on this plot with 16X sequencing depth being the previously established^17^ threshold for the depletion data (black dotted line) and the second and third standard deviations from the mean being the thresholds for the capture data (green and blue dotted lines, respectively).

The two datasets being compared differ in two important ways: (a) absolute sequencing depth (where capture $\gg$ depletion) and (b) the ratio of the sequencing depth for a *Wolbachia* sequence found as a nuwt relative to the same sequence found in the endosymbiont genome (where depletion $\gg$ capture) (Fig. 1). More specifically, the entire 90 Mbp *Brugia* genome was sequenced in the depletion dataset to a sequencing depth of only 100–150X which is lower than the 2000–3000X sequencing depth in the capture dataset across the *Wolbachia* genome. A direct comparison of the coverage on the *Brugia* genome or the *Wolbachia* genome is not possible given that the depletion data has the *Wolbachia* endosymbiont sequencing reads significantly reduced while the capture data has the *Brugia* sequencing reads reduced. In the depletion data, since few reads are derived from the *Wolbachia* endosymbiont genome, when all reads are mapped to the endosymbiont genome, we expect clearly delineated peaks in the sequencing depth that correspond to nuwts and the height of the peak is expected to be proportional to the number of copies of that nuwt (Fig. 1A). In the capture data we expect these peaks still occur, but they are added to a background sequencing depth from the reads derived from the *Wolbachia* endosymbiont genome (Fig. 1B). As a result of these two factors, the capture data has a much larger sequencing depth, but the antibiotic depletion of *Wolbachia* in the depletion data leads to higher ratio differences in sequencing depth. Despite these differences, the sequencing depth for each nt position in the *w*Bm genome correlates well between the capture data and the depletion data (Fig. 2, p-value: <2e-16, R-squared = 0.307). Therefore, we expect that regions of increased sequencing depth in the capture data correspond to multiple copies of *Wolbachia* sequences present in the host’s genome as multi-copy nuwts.

While a correlation is observed between the sequencing depth values for the capture data and those for the depletion data, as described above, such a correlation does not necessarily imply that sequencing depth can accurately predict nuwts. There are three factors that will influence the ability to predict nuwts using the capture data: (1) the copy number of the nuwt, (2) the relative abundance of *Wolbachia* chromosomes compared to the nematode chromosomes, and (3) the presence of suitable oligonucleotides in the capture pool. Factors 1 and 2 are interdependent. For example, if there are four *Wolbachia* genome equivalents for every nematode genome equivalent in the sample, then it may only be possible to predict nuwts that have at least four copies per nuclear genome, resulting in a 2-fold increase in sequencing depth for diploid organisms. However, the third factor is independent as it relates to how the oligonucleotide capture pool was designed. In this case, the major concern is the known removal from the capture pool of baits corresponding to reference nematode sequences, which included some nuwts. The exclusion of nuwts will lead to gaps in the tiling of the *Wolbachia* genome, although these missing regions may be partially or completely captured by probes to adjacent sequences in a fragment. With regards to all three of these factors, we expect this depletion data set to better predict nuwts than this capture data set. However, as discussed in the Background, Wo*lbachia-*depletion is not always feasible.

**Figure 3:**
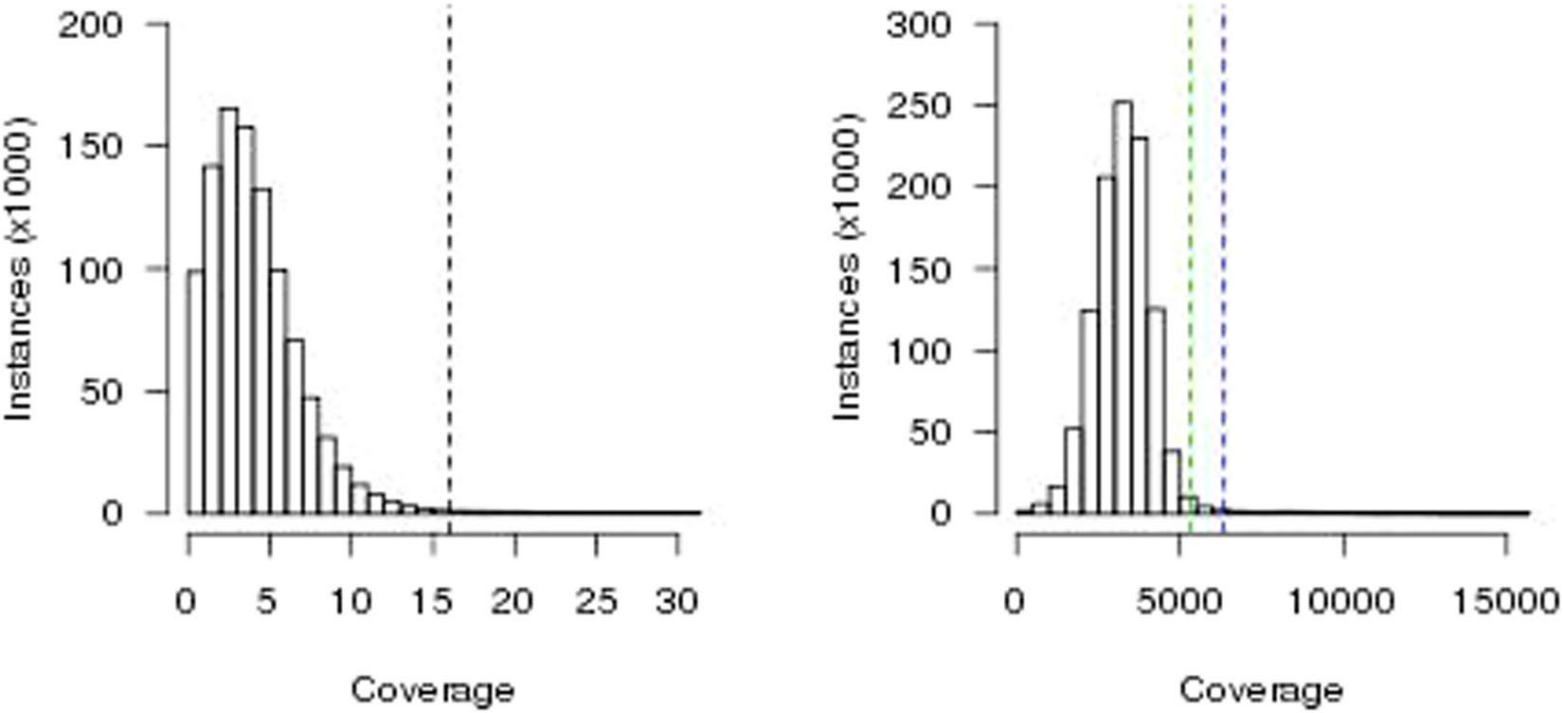
Sequencing Depth Histograms. Histograms of the instances, in increments of 1000, of a given sequencing depth upon calculating sequencing depth for every position in the genome are shown for (**A**) the depletion data published in Ioannidis *et al*.^17^ and (**B**) the capture data published in Geniez *et al*.^31^. In panel A, the previously established^17^ threshold for predicting a nuwt in the depletion data is shown with a dashed black line. In panel B, the second and third standard deviations from the mean are shown with dashed green and blue lines, respectively.

Previously^17^, we described the limits of applying statistical tests and models to predict nuwts given that the sequencing depth distribution of the depletion data was not bell-shaped and did not have a unimodal distribution (Fig. 3A, Table 1). In that study, for detection of nuwts, we were able to justify an empirically derived threshold of 16x sequencing depth through visual comparison of the sequencing depth distributions of the *B. malayi* sequencing data and the *Wolbachia* data^17^. With the oligonucleotide capture system presented here, sequencing data from the *B. malayi* genome lacking homology to *Wolbachia* sequence is not available for such an analysis. However, the sequencing depth distribution of the capture data appears to have a bell-shaped distribution (Fig. 3B) with similar values for the mean and median suggesting it is unimodal, or nearly unimodal (Table 1). This is most likely the result of the increased sequencing depth across the entire *Wolbachia* genome and the smaller relative difference in sequencing depth between any single copy *Wolbachia* genes and any single copy nuwts. Given that the sequencing depth distribution appears to be bell-shaped, we can use the standard deviation to set a threshold for distinguishing nuwts. Using two or three standard deviations, the threshold for detecting nuwts would be 5327x or 6348x, respectively (Fig. 3B).

**Table 1:** Sequencing Statistics for the Capture and Depletion Data Sets.

	Depletion (SRX142902)	Capture (SRX1057997)
Million read pairs sequenced	69.3	45.9
Bases (Gbp)	13.7	4.6
Read length (bp)	99	50
Median insert size ± absolute deviation (bp)	206 ± 39	180 ± 44
Reads pairs mapped to *Wolbachia* genome	76,596	35,843,198
Mean sequencing depth ± standard deviation	13.0 ± 65.1	3286 ± 1020
Median sequencing depth	4	3271
Maximum sequencing depth	1,613	14,305

Figure 2 can then be divided into quadrants based on these thresholds and the one previously established for the depletion data. The upper right quadrant (UR) will contain the data for nuwts that were predicted with both datasets while the lower left (LL) quadrant contains the data for regions predicted to not include nuwts for both datasets. The remainder of the data points are found in the upper left quadrant (UL) and lower right quadrant (LR). The UL should contain regions only predicted as nuwts using the *Wolbachia*-enriched capture data while the LR contains nuwts only predicted using the *Wolbachia*-depleted data. Of note, the three factors identified above, which could influence our predictions (i.e. nuwt copy number, *Wolbachia*-nematode relative abundance, and suitable probes), will all lead to data appearing in the LR; we could not identify any factors that would lead to data appearing in the UL.

To examine how the threshold for predicting nuwts effects our observations and the placement of data in these three quadrants, we sought to examine the difference between using two and three standard deviations as a threshold cutoff for the capture data. If three standard deviations are used yielding a threshold of 6348x, then 1,042,854 of 1,080,085 genomic positions (96.6%) are predicted in the same manner between the two datasets. However, while 49,049 nucleotide positions were predicted as being in nuwts in the depletion data, only 12,122 such positions are predicted with the capture data. This suggests that a three standard deviation threshold may be too stringent. By comparison, if two standard deviations are used, yielding a threshold of 5327x, then 1,045,613 of 1,080,085 positions (96.8%) are predicted in the same manner with the two datasets. However, while 49,049 nucleotide positions were predicted as being in nuwts in the depletion data, 20,861 such positions are predicted with the capture data. Therefore, while the lower threshold increases the overall similar predictions by only a small percentage (96.7% v. 96.9%), it nearly doubles the number of predictions of nucleotides in nuwts that are similar to the depletion data (24.7% v. 42.5%). This might argue in favor of the less stringent threshold.

However, a different picture emerges upon examining the quadrants. The more stringent three standard deviation threshold places 37,079 points in the LR quadrant, whereas the less stringent threshold places 31,330 points in this area (Table 2). However, the number of predictions in the UL increases almost 20-fold, going from 152 with the more stringent threshold to 3,142 with the less stringent threshold (Table 2). Data points in the UL are not easily attributed to any given factor of the experimental design, so it might be desirable to minimize them, arguing in favor of the more stringent threshold. Clearly there are trade-offs that need to be balanced in assigning the threshold. Ultimately, the best threshold employed should be based on the hypothesis being tested and further downstream analyses that are undertaken, including the manner in which the predictions are validated.

**Table 2:** *Wolbachia* Sequencing Depth Threshold Analysis.

	Positions with ≥16X Sequencing Depth in Depletion Data	Positions with <16X Sequencing Depth Depletion Data
Positions with ≥6348X in the Capture Data	11,970	152
Positions with <6348X in the Capture Data	37,079	1,030,884
Positions with ≥5327X in the Capture Data	17,719	3,142
Positions with <5327X in the Capture Data	31,330	1,027,894

### Contribution of factors resulting in under-identifying nuwts

As described above, we expected to have points in the LR quadrant due to three factors. The third factor, that the oligonucleotide capture design did not include some nuwt sequences, would result in nuwts predicted by the depletion data, but having very low sequencing depth in the capture data. There are 13,839 data points that are more than two standard deviations below the mean in the capture data, or <1246x sequencing depth, suggesting absence of a probe (factor 3). Of those, ~16%, or 2,225 data points are from regions above the threshold for predicting nuwts using the depletion data, meaning the two data sets are in conflict. These positions were found to occur in 14 regions of the *Wolbachia* genome. For all of these regions, we confirmed that probes had been removed from the capture design due to homology to a nuwt in a filarial nematode genome. Therefore, the decrease in sequencing depth is likely due to the absence of suitable probes in the capture design.

### SNPs identify LGTs

We expect the sequence reads mapping to *w*Bm in the capture data to contain some proportion of SNPs, given that both nuwts and the endosymbiont genome will have accumulated mutations following transfer, particularly nuwts that are no longer under selection. In some cases, the base call for the endosymbiont genome may not even be the dominant base call due to the high copy number of some nuwts in the *B. malayi* genome^17^. There are four factors that influence the presence and extent of variation in the sequence reads: (a) differences between the endosymbiont consensus genome being sequenced and the reference endosymbiont genome, (b) heterogeneity in the genome of the *Wolbachia* endosymbiont population sequenced, (c) the presence of a SNP in the nuwt at a detectable level, and (d) the presence of SNPs in nuwt paralogs.

Differences between the endosymbiont genome queried and the reference genome sequenced would be observed as homogenous variation that is present in almost 100% of the underlying reads. Across the entire reference 1.08 Mbp *w*Bm genome^34^, there are 17 nt positions (0.0016%) where >99.9% of the sequencing reads obtained from the oligonucleotide capture system support an alternate base call relative to the reference *w*Bm genome. Eight of these positions had an alternate base call supported by 100% of reads while nine positions had an alternate base call supported by >99% of the reads, indicative of a sequencing error in a single read. This indicates that either (a) there are sequencing errors in the reference genome at these 17 positions or (b) there is sequence variation between the endosymbionts used to generate the oligonucleotide capture data set and those used to generate the BACs for sequencing the reference *w*Bm genome. These positions are located in six regions of the genome, including in a portion of an intergenic region at *w*Bm coordinates 678, 738–678, 827; a tryptophanyl-tRNA synthetase at 692, 659–692, 669; a 4-hydroxybenzoate polyprenyltransferase at 695, 065–695, 066; a region containing nine putative genes at 832, 790–838, 394; DNA polymerase I at 948, 208–949, 008; and a hypothetical protein at 951,478. This analysis is not possible for the antibiotic-depleted *B. malayi* since the non-nuwt regions on the wBm genome typically had <5X sequencing depth.

The other three factors (i.e. *Wolbachia* population heterogeneity within the nematodes sequenced, nuwt SNPs, and nuwt paralog SNPs) would result in heterogeneous variation at a specific position. In the case of variation in the *Wolbachia* endosymbiont population, the position should have total sequence read depth similar to the mean. Using either the second or third deviations above the mean as a cutoff, as described above, we find a higher proportion of positions with >20% variation in the underlying reads for putative nuwts with increased sequencing depth than those regions with sequencing depth values predictive of placement in the endosymbiont genome. For the positions with sequencing depth values within two standard deviations of the mean, we find only 0.06% have >20% variation in the underlying reads, a number that may be artificially inflated since nuwts are under-predicted using either of these standard deviation thresholds. In comparison, for regions predicted to contain nuwts with either the two standard deviation or the three standard deviation threshold we find 1.8% of positions have >20% variation in the underlying reads. For the 12,122 positions that have capture data with ≥6,348x sequencing depth, 121 positions have >50% of the reads underlying that position supporting a variant base, meaning a majority of the reads support an alternate base call relative to the reference. Yet the reference base call was always supported by >18% of the underlying capture reads for these 12,122 positions. This suggests that the reference base call was correct but that a majority of the sequence arose from higher sequencing depth of *Wolbachia* sequences from the nuclear genome.

We also sought to examine the converse – if genetically variable positions had increased sequencing depth. There are 1,020 positions with >20% variation in the underlying reads. These positions have an average sequencing depth of 5139X with a standard deviation of 1976X with values ranging from 324X to 12,865X and a median sequencing depth of 4849X. Since sequencing depth of 5327x corresponds to 2 standard deviations above the mean sequencing depth, it follows that nearly half of the genetically variable sites had a sequencing depth value that was more than 2 standard deviations higher than the average sequencing depth across the genome. As such, it is likely that SNPs alone can be used to predict some nuwts in at least some hosts of *Wolbachia* endosymbionts.

### Comparison with prior validation results

Nuwts in *B. malayi* have been described and validated twice previously^7, 17^. The first validation was of a subset of nuwts detected in the assembled *B. malayi* genome^7^. These nuwts were validated by PCR amplification of the junctions of nuwts and the nuclear genome followed by end sequencing verification of the products. Unfortunately, given that the probe set used here had probes removed that had homology to the filarial nematode genome, we anticipate that those nuwts would be poorly recovered with uneven results. Consistent with this, the 12 genes that were expected to be found in these amplification products had sequencing depth ranging from 754x (low sequencing depth) to 6275x (~3 standard deviations above the mean sequencing depth).

**Figure 4:**
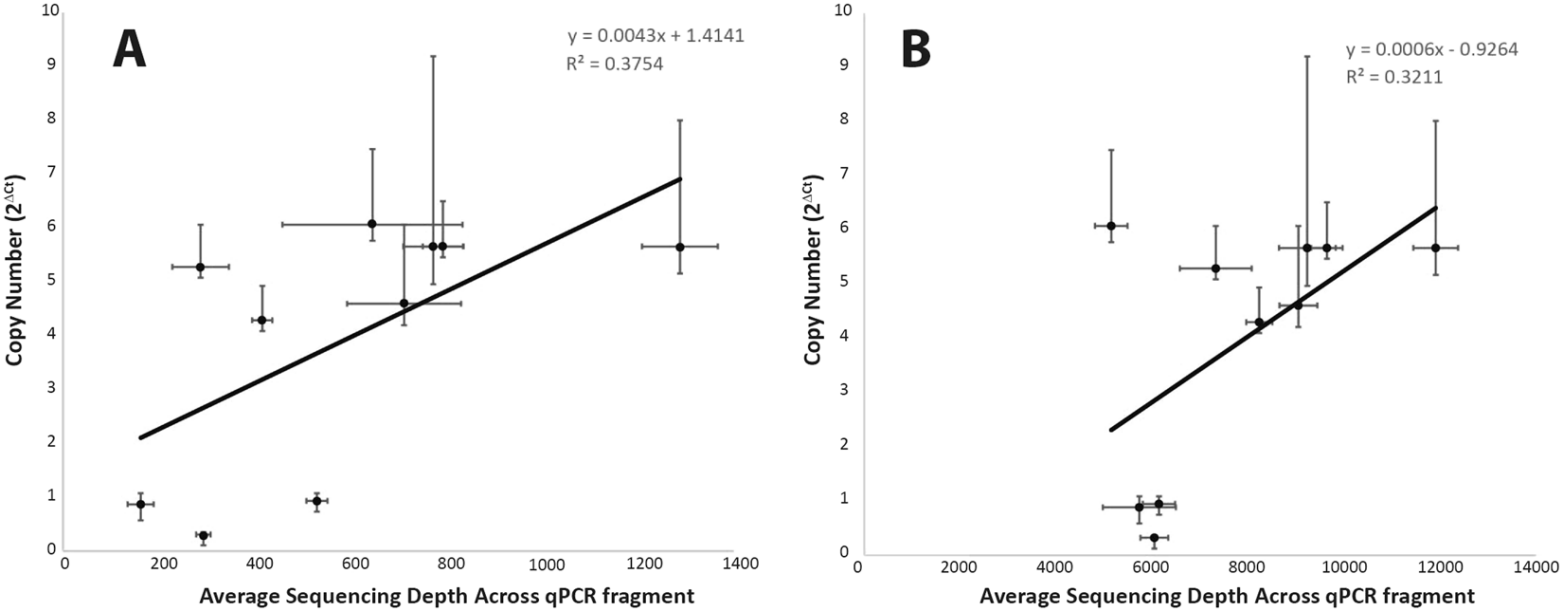
Correlation Between Previous qPCR Results and Sequencing Depth of the Capture Data. For ten genes, the average sequencing depth across a fragment amplified by qPCR is compared to the previously measured^17^ copy number, measured by the ΔCt of the qPCR reaction relative to the average Ct value of six single copy *B. malayi* genes, for the depletion data^17^ (Panel A,) and the capture data (Panel B). The error bars for the copy number are derived from one standard deviation of the ΔCt, making them asymmetric since copy number is exponentially related to ΔCt. The error bars for the average sequencing depth are one standard deviation. A comparison of the plots for the capture data presented here and the previously presented depletion data reveals similar R^2^ values but different y-intercepts and slopes. A lower y-intercept is expected for the capture data relative to the depletion data since it will be altered by the presence of the sequencing reads from the endosymbiont genome. A different slope is expected due to the differences in the sequencing depth.

The second validation of nuwts was conducted on nuwts detected using sequencing depth and SNPs in the previously published depletion data^17^ that is used for comparisons here. Validation was conducted by using qPCR to measure the copy number of nuwts for comparison to the sequencing depth, which should also reflect the copy number^17^. A comparison of this previous qPCR data with the capture data presented here reveals similar R^2^ values for both data sets, suggesting that at least a subset of nuwts can be predicted accurately (Fig. 4). The plots reveal different y-intercepts, which is expected given the differing presence of sequencing reads from the endosymbiont genome between the two data sets (Fig. 4). The slopes are different, reflecting different levels of sequencing depth (Fig. 4).

## Discussion

### Identifying nuwts using capture-based sequencing

Numerous LGTs from *Wolbachia* to its many hosts have been described, which are relatively recent in nature^7, 8, 11, 12, 14, 15, 17^. Evidence for most of these suggests that they are evolving neutrally, accumulating mutations at a slow rate, including deleterious mutations^7, 8, 11, 12, 14, 16^. Most of the sequences are still very recognizable using BLASTN-based searches or using mapping algorithms like BWA^35^, BOWTIE^36, 37^, MOSAIK^38^, and STAMPY^39^. Therefore, it is not surprising that they are also captured using an oligonucleotide-based approach. Using two existing datasets, we demonstrate that nuwts can be identified using sequence data following selection with an oligonucleotide-based capture system even in the presence of the endosymbiont genome.

However, fewer nuwts were detected in the capture data than in the depletion data likely owing to a combination of factors including that (a) the oligonucleotides for capture design excluded some nuwts in the reference *B. malayi* genome and (b) the dynamic range between sequencing depth of the endosymbiont genome and nuwts is smaller. Absence of some nuwt sequences from the oligonucleotide bait pool used for capture appears to account for ~6.0–7.1% of the missing data points, depending on which threshold is applied. This has been corrected with the most recent capture design by adding back regions of the *Wolbachia* genome that were removed due to similarity with nematode genomes. Although certain nuwts were under-represented, the capture approach nonetheless provided sequence of the *Wolbachia* genome^31^, suggesting that adjacent baits can capture these missing sequences, albeit at lower sequencing depth levels.

**Figure 5:**
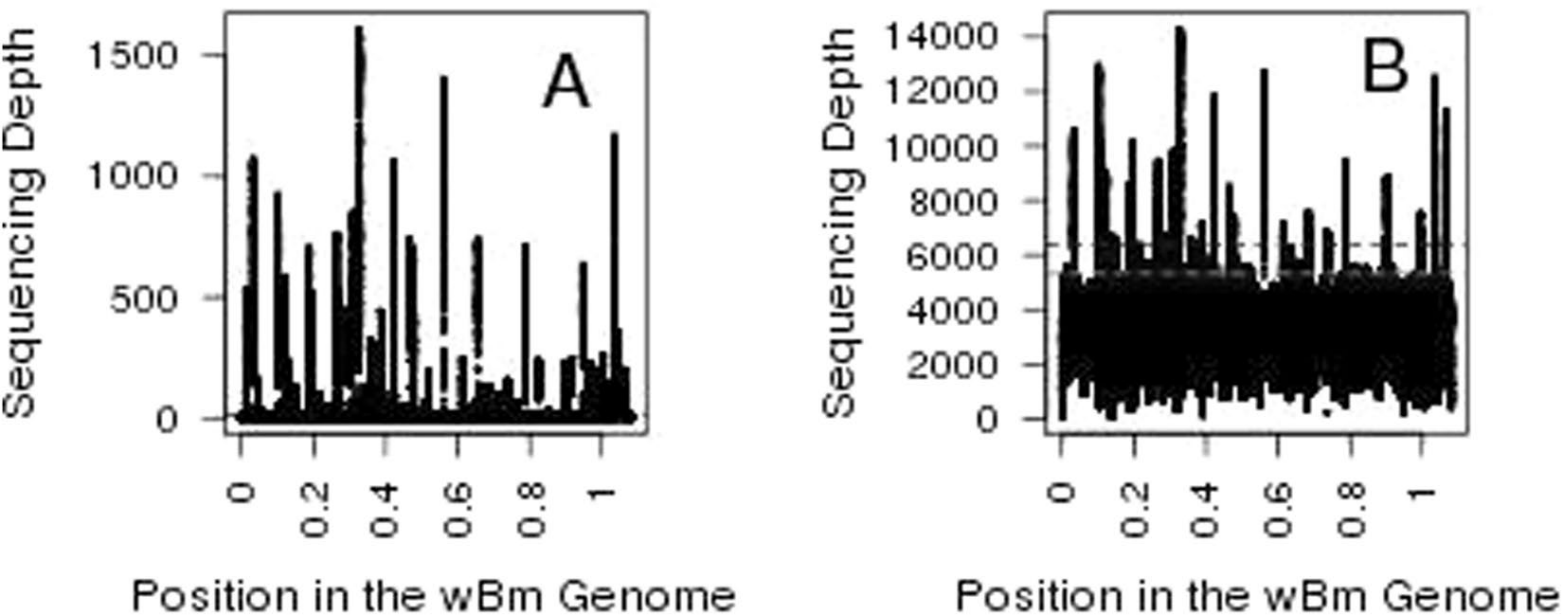
Sequencing Depth Across the *w*Bm genome. The sequencing depth is plotted for each position along the *w*Bm genome for the depletion data (Panel A) and the capture data (Panel B). Multi-copy nuwts are the highest peaks in sequencing depth. The thresholds for detecting sequencing depth are overlaid (16-fold cutoff for depletion data, red dotted line in Panel A, and two and three standard deviation cutoffs in the capture data, green and blue dotted lines, respectively, in Panel B). The dynamic range between the sequencing depth across multi-copy nuwts relative to the average sequencing depth across the genome is lower in the capture data than in the depletion data. Where the dynamic range is larger, as in the depletion data, it is much easier to detect nuwts, particularly lower copy number nuwts, demonstrating the utility of depleting of *Wolbachia* when possible.

The differences between these datasets and the ability to detect nuwts with these datasets highlights that there is not a single method that can be applied to appropriately detect nuwts in all datasets. The appropriate method will always be related to the sequencing technology, the relative proportion of *Wolbachia* and host reads, and the characteristics of the most closely related reference genomes. The copy number of the nuwts and the relative abundance of, and thus sequencing depth difference between, the *Wolbachia* and *B. malayi* chromosomes appear to be the dominant factors influencing misidentification of nuwts. Where the dynamic range of the ratio of the sequencing depth across multi-copy nuwts relative to the average sequencing depth across the genome is larger, as in the depletion data, it is much easier to detect nuwts (Fig. 5). Therefore, while nuwts can be predicted in the presence of the endosymbiont, it is better to predict nuwts following *Wolbachia* depletion when possible. When depletion is not possible, care must be taken to identify thresholds best suited to the data at hand including the ratio of sequencing depth or copy number between the nuclear genome and endosymbiont genome, as well as the evenness of sequencing depth, which can be influenced by factors like library construction.

Using the capture data, nuwts can be predicted using sequencing depth and SNP variation in the underlying sequence reads. While the best strategy and thresholds used likely depend on the nature of the data, it is likely that the combination of sequencing depth and genetic variation would produce the best result, and the union of the two predictions would produce the most conservative estimate of the true nuwt content in a genome. Such predictions could guide researchers interested in *Wolbachia-*host LGT to candidate regions of the genome to be examined with more focused approaches. Of course each prediction method has its own benefits and limitations, most notably the sole use of polymorphisms may prevent the detection of recent nuwts that have yet to accumulate mutations.

### Effect of nuwts on genome consensus of Wolbachia endosymbionts

Given that we have already shown that nuwts can be present in the *B. malayi* genome in many copies, it is not surprising that sometimes the reads derived from nuwts may outnumber the reads derived from the *Wolbachia* genome. When >50% of reads originate from a SNP-containing nuwt, the consensus genome sequence for the *Wolbachia* genome will be incorrect. This appears to have happened for 227 positions in the current data, which range in sequencing depth from 2546x to 12,865x. Of these, 17 SNPs were supported by ≥99.9% of the underlying reads, which may indicate either a sequence error in the reference, or a difference in this endosymbiont genome sequence relative to the reference.

The remaining 210 positions with intermediate levels of genetic variation (>50% and <90%) demonstrate that the sequencing depth and the polymorphisms arise from a contribution of the nuwts and the endosymbiont genome with the endosymbiont base being the minor variant. In these cases, a majority rules genome consensus calling algorithm is likely to yield an incorrect consensus endosymbiont genome. It is important to note that this is not a problem limited to capture-based sequencing. This will occur with any sequence-based method involving multiple alleles. In the filarial nematode system, it may be easier to detect because there are many relatively small nuwts (<10-kbp) with multiple copies allowing the use of both sequencing depth and polymorphism for detection. However, this will also be an issue in the insect systems and sequencing depth may prove more difficult to measure since much larger nuwts are typically observed. It might seem that the use of long reads, like those from the Pacific Biosciences RS, would remedy this problem. However, we have already observed this problem in at least one *Wolbachia* genome assembled using Pacific Biosciences data. These errors are likely dependent on the error-correction algorithms used. As such, as the technology develops and long, error-free reads can be obtained, it would likely eliminate these problems. Using a BAC-based sequencing approach to obtain endosymbiont genomes can alleviate this problem, as is the case with the reference *w*Bm genome. However, such BAC-based sequencing is no longer considered cost effective. In the absence of a BAC-based approach, care should be taken in assessing the functionality of genes, or the lack thereof, in systems where LGT to the host is likely. More specifically, studies examining pseudogenes in endosymbiont genomes and proposing the loss of functionality (e.g. refs 40–42) need sufficient experimental validation of the responsible SNPs to ensure their location in the endosymbiont genome, since any analogous nuwts are likely to be pseudogenized.

## Conclusions

DNA from *Wolbachia* is found in the genomes of a great number of its hosts as nuwts. The detection of nuwts is often linked to careful analyses of the host genome sequencing project. Low-cost methods to detect and sequence nuwts are needed in order to better understand the extent of such transfers in host genomes. Such studies lay the groundwork for how such integrations occur and if they have any functional significance. Here, we demonstrate that oligonucleotide-based capture systems can be used to capture and sequence nuwt sequences. Such sequences can be distinguished from the bacterial endosymbiont genome by an increase in sequencing depth as well as genetic heterogeneity of the sequences. However, the predictions are likely to be more limited than when a depletion based strategy can be employed. Lastly, we demonstrate that the presence of nuwts can confound genome consensus calling, yielding an erroneous genome sequence of the endosymbiont.

## Methods

### Data sets

This manuscript compares two Illumina paired end datasets. The first data set, which is referred to as the depletion data, is described by Ioannidis *et al*.^17^ (SRX142902). This data set includes >138 million reads from a 300-bp paired end library constructed directly from DNA from tetracycline-treated, and thus *Wolbachia-*depleted, *B. malayi* filarial nematodes. The second data set, which is referred to as the capture data, is described by Geniez *et al*.^31^ (SRX1057997). This data set includes >91 million reads from a paired end library constructed from *B. malayi* DNA library from which *Wolbachia*-derived sequences were captured using the Agilent Sure Select protocol and RNA baits as previously described^31^.

### Sequence alignments

For the depletion data set, the alignments from Ioannidis *et al*.^17^ were used, which were aligned with BWA version 0.5.9-r16^35^ to the reference *w*Bm genome (AE017321.1)^34^ with default parameters and had duplicates removed with MarkDuplicates as implemented in Picard 1.48^43^. To ensure comparability of the data, the capture data was re-aligned with BWA version 0.5.9-r16^35^ with default parameters to the reference *w*Bm genome (AE017321.1)^34^ and had duplicates removed with MarkDuplicates as implemented in Picard version 1.48^43^. Statisics about both libraries and sequencing were collected with Picard version 1.48^43^.

### Sequencing depth, SNP identification, and other calculations

The sequencing depth of the sequences was measured with MPILEUP in SAMTOOLS version 0.1.19-44428cd^44^ using the alignments described above. SNPs were identified using this MPILEUP output. PERL version 5.8.8 and LINUX commands (e.g. CUT, AWK, and SED) were used to parse data and perform the calculations presented. Figures were constructed and statistical tests performed in R version 2.15.2.
